# Impact of host intraspecies genetic variation, diet, and age on bacterial and fungal intestinal microbiota in tigers

**DOI:** 10.1002/mbo3.1050

**Published:** 2020-05-12

**Authors:** Haiying Jiang, Wu Chen, Li Su, Mingwei Huang, Libo Lin, Qiao Su, Guanyu Li, Hafiz Ishfaq Ahmad, Linmiao Li, Xiujuan Zhang, Huiming Li, Jinping Chen

**Affiliations:** ^1^ Guangdong Key Laboratory of Animal Conservation and Resource Utilization Guangdong Public Laboratory of Wild Animal Conservation and Utilization Guangdong Institute of Applied Biological Resources Guangzhou Guangdong China; ^2^ Guangzhou Zoo Guangzhou Guangdong China; ^3^ Zhongkai University of Agriculture and Engineering Guangzhou Guangdong China; ^4^ The First Affiliated Hospital of Sun Yat‐sen University Guangzhou Guangdong China

**Keywords:** age, diet, gut microbiome, mycobiome, subspecies, tiger

## Abstract

The bacterial microbiota in the gut varies among species, as well as with habitat, diet, age, and other factors. Intestinal microbiota homeostasis allows a host to adjust metabolic and immune performances in response to environmental changes. Therefore, potential implications of the gut microbiota in sustaining the health of the host have gained increasing attention in the field of endangered animal conservation. However, the effect of host intraspecies genetic variation on the gut microbiota is unknown. Moreover, little is known about the complexity of the gut mycobiota. Tigers are listed as endangered species, raising worldwide concern. Potential influences of subspecies, diet, and age on the gut microbiota in tigers were investigated in this study to provide a better understanding of the response of the tiger gut microbiota to external changes. The results revealed that the impacts of the factors listed above on gut bacterial and fungal communities are versatile. Host intraspecies genetic variation significantly impacted only fungal alpha diversity of the gut microbiota. Differences in diet, on the other hand, had a significant impact on alpha diversity of the gut microbiota, but exerted different effects on beta diversity of gut bacterial and fungal communities. Host age had no significant impact on the diversity of the gut fungal communities, but significantly impacted beta diversity of gut bacterial communities. This comprehensive study of tiger gut microbiota is an essential reference for tiger conservation when considering feeding and management strategies, and will contribute to a better understanding of the mycobiota in wildlife.

## Graphical abstract

A potential influence of subspecies, diet, and age on the gut microbiota in tigers was investigated in this study to provide a better understanding of the response of the tiger gut microbiota to external changes. The results revealed that the impact of these factors on gut bacterial and fungal communities are versatile.

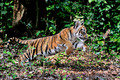

## 1 INTRODUCTION

Many investigations have revealed that the gut microbiota plays an important role in host health, such as the modulation of the host immune system, while also affecting host development and physiology (Sommer & Bäckhed, 2013). Dysbiosis in gut microbiota is associated with many diseases, such as metabolic diseases and neurodevelopmental disorders (Marchesi et al., 2016). The gut microbiome fluctuates with changes in external factors, such as habitat, diet, disease, and medication. For example, antibiotic treatment, cesarean section, and formula milk feeding contribute to delayed microbiome development and altered alpha diversity (Bokulich et al., 2016). Moreover, gut microbiota can be modulated by dietary changes (Johnson et al., 2019; Youngblut et al., 2019). Upon habitat changes, wild animals were found to display differences in microbial community composition and bacterial diversity (Huang, Chang, Huang, Gao, & Liao, 2018; Watson et al., 2019). The gut microbiota is known to vary because of host genetic variation above the species level in mammals (Ley et al., 2008; Nishida & Ochman, 2017; Song et al., 2020), but the effect of intraspecies differences on the gut microbiota is still unknown. Microbiome fluctuations allow the host to adjust metabolic and immune performances in response to environmental changes (Candela, Biagi, Maccaferri, Turroni, & Brigidi, 2012). Fecal microbiota transplantation has been used in humans to treat diseases, such as *Clostridium difficile* infection, irritable bowel syndrome, and inflammatory bowel diseases (Bajaj et al., 2017; Kelly et al., 2015).

So far, investigators have mostly focused on the bacteria present in the gut. Other microorganisms that are less common are often overlooked, such as archaea, viruses, and microbial eukaryotes (Cotter, Huseyin, Scanlan, & O’Toole, 2017). In recent years, fungi have increasingly been recognized as defining constituents of the gut microbiota, cofactors in disease, and interaction partners of the immune system (Huffnagle & Noverr, 2013; Sam, Chang, & Chai, 2017; Underhill & Iliev, 2014; Wheeler et al., 2016). Moreover, the gut mycobiome may act as a reservoir for opportunistic pathogens in immunocompromised hosts (Polvi, Li, O’Meara, Leach, & Cowen, 2015). An earlier report showed that fungi in the gut were stable over time (Scanlan & Marchesi, 2008), whereas other reports indicated that the gut mycobiota was transient and lacked core species (Hallen‐Adams, Kachman, Kim, Legge, & Martínez, 2015). Studies in humans and mice have implied that the dynamics of mycobiota in the gut are influenced by the underlying pathophysiology and environment (Dollive et al., 2013; Iliev et al., 2012). However, studies on the fungal microbiota are still rare and focus mainly on humans. Little is known about how the gut mycobiota is influenced by various factors, and whether these factors exert similar effects on the fungal and bacterial intestinal microbiota.

The potential implications of the gut microbiome in endangered animal conservation have increasingly been drawn attention to in recent years, and a new branch of conservation biology, that is, conservation metagenomics, has been proposed (Stumpf et al., 2016; Wei et al., 2019; West et al., 2019). A total of 13,482 animal species worldwide are currently threatened with extinction, including vulnerable, endangered, and critically endangered species, according to the IUCN Red List of Threatened Species version 2018‐2. More and more threatened species have been brought under intensive management or into captivity to save these animals. However, captive programs are often impeded by disease epidemics and poor health, as well as lower reproductive rates and higher offspring mortality compared to their wild‐born counterparts (Jiang et al., 2017; Lowenstine, McManamon, & Terio, 2015; Seimon et al., 2013; Wasimuddin et al., 2017; Yang et al., 2017; Zhao et al., 2017). During intensive management or captivity, animals experience a range of changes regarding diet, drug treatment, and habitat, which may significantly alter the gut microbiome of these threatened animals. Numerous studies have already recorded the changes in the gut microbiome between captive and wild populations of endangered animals, such as those observed in dugongs (*Dugong dugon*), black howler monkeys (*Alouatta pigra*), nonhuman primates, Australian sea lions (*Neophoca cinerea*), and crocodile lizards (Amato et al., 2013; Clayton et al., 2016; Delport, Power, Harcourt, Webster, & Tetu, 2016; Eigeland et al., 2012; Jiang et al., 2017). A detailed understanding of the gut microbiome will have important implications for the management of endangered animal health in captivity.

The tiger (*Panthera tigris*) is one of the largest Felidae species, with six extant subspecies (Amur, northern Indochinese, Malayan, Sumatran, Bengal, and South China [SouthCN] tigers), and three extinct subspecies (Bali, Javan, and Caspian tigers) (Luo et al., 2004). Tigers are classified as endangered, with a decreasing population trend (Goodrich et al., 2015), and are further listed in the Convention on International Trade in Endangered Species of Wild Fauna and Flora (CITES) Appendix I. The total number of mature tigers worldwide was only 2,154–3,159 in 2014 (Goodrich et al., 2015). Moreover, SouthCN tigers have not been directly observed in the wild since the 1970s and are possibly extinct in the wild. Only 178 SouthCN tigers are conserved in zoos. Tiger cubs depend on artificial feeding, because cubs nursed by their mothers show low survival rates. In addition, the mother tigers are often unwilling to take care of their cubs when kept in zoos (Li, Lei, & Chen, 2013). Tiger cubs often die of indigestion and gastrointestinal diseases during artificial feeding (Li et al., 2013). Therefore, a detailed understanding of the gut microbiota of tigers will prove important for raising tiger cubs and to improve survival rates.

This study was designed to investigate the intestinal microbiota in healthy tigers, and the impact of host genetic variation within species (subspecies and inheritance), diet, and age on the gut microbiota, to provide a better understanding of the gut microbiome, reduce the knowledge gap concerning fungal intestinal communities, and provide support for healthy tiger management. Bacterial and fungal microbial communities were studied to determine whether the effects of the above factors on the gut microbiota were the same for both communities.

## 2 MATERIALS AND METHODS

### 2.1 Sample collection

All tigers were housed at Guangzhou Zoo (China). When fresh feces of tigers were observed, sterile swabs were used to collect the feces from the center of the dunghill. These samples were placed in sterile tubes, immediately frozen in liquid nitrogen, and then transported to the laboratory for DNA extraction.

A total of 24 tigers were sampled, including 19 adults or subadults (3–16 years old), and 5 cubs (no more than 6 months old). Adult and subadult animals were fed meat, more specifically, a mixture of lean meat, chicken, and beef at a fixed ratio. Cubs were fed milk (KMR^®^ kitten milk replacer powder, Pet‐Ag, Inc.) before 30–40 days of age; 40‐ to 60‐day‐old cubs were fed a mixture of milk and meat and eventually changed to the full meat diet at 2–3 months old. Probiotics Sachet Children's Formula (Health and Happiness [H&H] International Holdings Limited) and digesting enzymes were added to the milk. Collected samples were used to study the effects of host genetic variation (reflected by the effects of subspecies and inheritance), diet, and age on the gut microbiota in tigers. Detailed sample information is shown in Table 1.

**Table 1:** Sample information

Sample	Sex	Subspecies	Age	Diet	Sampling date	Group			
						Subspecies	Diet	Age	Heredity
BAI1	M	Bengal	7 years	Meat	2017.12.21	Bengal			
NAN7	F	Bengal	9 years	Meat	2017.12.21	Bengal			
DONG1	M	Bengal	16 years	Meat	2017.12.22	Bengal			P3
JING1*	F	Bengal	15 years	Meat	2017.12.22	Bengal			P3
JING2#	F	Bengal	15 years	Meat	2017.12.22	Bengal			P3
W3*	M	Bengal	5 years	Meat	2017.12.23	Bengal			F3
W4#	M	Bengal	5 years	Meat	2017.12.23	Bengal			F3
ZHEN1	F	Bengal	7 years	Meat	2017.12.22	Bengal			
DMEN6	M	Amur	8 years	Meat	2017.12.21	Amur			P4
GUO1	F	Amur	3 years	Meat	2017.12.21	Amur			F4
SYI13	F	Amur	8 years	Meat	2017.12.22	Amur			P4
SYIN4*	F	Amur	8 years	Meat	2017.12.20	Amur			
SYINd#	F	Amur	8 years	Meat	2017.12.23	Amur			
XMEN6	M	Amur	8 years	Meat	2017.12.21	Amur			
YIYI12	M	Amur	7 years	Meat	2017.12.21	Amur			
AN1	F	SouthCN	4 years	Meat	2017.11.06	SouthCN		Adult	
HUI12	F	SouthCN	6 years	Meat	2017.11.26	SouthCN		Adult	P1
KK1*	M	SouthCN	8 years	Meat	2017.11.04	SouthCN		Adult	P2
Kkaa#	M	SouthCN	8 years	Meat	2017.12.05	SouthCN		Adult	P2
LE1	F	SouthCN	6 years	Meat	2017.11.13	SouthCN		Adult	P2
TO5	F	SouthCN	10 years	Meat	2017.12.15	SouthCN		Adult	
TT8	M	SouthCN	5 years	Meat	2017.12.16	SouthCN		Adult	
YY13	M	SouthCN	6 years	Meat	2017.11.26	SouthCN		Adult	P1
DB3	F	SouthCN	3 months	Meat	2018.02.03		Meat	Young	F1
DC7	M	SouthCN	2 months	Meat	2017.11.06		Meat	Young	F2
EC5	M	SouthCN	2 months	Meat	2017.11.06		Meat	Young	F2
HUAN3	F	SouthCN	6 months	Meat	2017.12.10		Meat	Young	F2
XB1	M	SouthCN	3 months	Meat	2018.02.03		Meat	Young	F1
DB13	F	SouthCN	50 days	Mix	2017.11.24		Mix		
DB20	F	SouthCN	62 days	Mix	2017.12.06		Mix		
DB25	F	SouthCN	70 days	Mix	2017.12.14		Mix		
EB13	M	SouthCN	50 days	Mix	2017.11.24		Mix		
EB20	M	SouthCN	62 days	Mix	2017.12.06		Mix		
EB25	M	SouthCN	70 days	Mix	2017.12.14		Mix		
DB9#	F	SouthCN	44 days	Mix	2017.11.18		Mix		
EB9#	M	SouthCN	47 days	Mix	2017.11.21		Mix		
DB33*	F	SouthCN	32 days	Milk	2017.11.06		Milk		
DB7*	F	SouthCN	42 days	Milk	2017.11.16		Milk		
EB3*	M	SouthCN	32 days	Milk	2017.11.06		Milk		
EB7*	M	SouthCN	42 days	Milk	2017.11.16		Milk		

Note

Samples sequenced in 16S and ITS1 rDNA analyses are not marked with any label. Samples sequenced for detecting only 16S and ITS1 rDNA are marked with a * and #, respectively. South China, SouthCN.

### 2.2 DNA extraction and sequencing

Total DNA was extracted from fecal samples using the PowerSoil^®^ DNA Isolation Kit (MOBIO Laboratories, Inc.). For bacterial community analysis, the V3–V4 hypervariable region of the 16S rRNA gene was amplified with 338F (5′‐ACTCCTACGGGAGGCAGCA‐3′) and 806R (5′‐GGACTACHVGGGTWTCTAAT‐3′) primers. For fungal community analysis, the first nuclear ribosomal internal transcribed spacer region (ITS1) was amplified using ITS1F (5′‐CTTGGTCATTTAGAGGAAGTAA‐3′) and ITS2 (5′‐GCTGCGTTCTTCATCGATGC‐3′) primers. High‐throughput sequencing was performed using the Illumina HiSeq 2500 platform (250 bp paired‐end reads) at Biomarker Corporation (Beijing, China).

After DNA extraction and amplification, a total of 34 samples were sequenced for bacterial community analysis. To study the effect of subspecies, the samples were grouped into three groups: Bengal, Amur, and SouthCN tigers. To study the effect of inheritance, four meat‐fed tiger families were studied (parents were marked as P1–P4, and correspondingly, offspring were marked as F1–F4). For studies on the effect of diet, samples were grouped into three groups: meat (meat‐fed tigers), milk (milk‐fed tigers), and mix (tigers fed a mixture of meat and milk). For the study on the effect of age, the samples were separated into two groups: adult (tigers aged 3–16 years) and young tigers (tiger cubs at ≤6 months old). First, all samples were grouped according to one factor, while ignoring the effects of the other two factors to provide an overview of the effect of each factor. Then, the samples were regrouped to exclude any effects caused by the other two factors. When the effect of the subspecies was studied, only samples from adult meat‐fed tigers were selected: *N*(Bengal) = 6, *N*(Amur) = 6, and *N*(SouthCN) = 7. For analyzing the effects exerted by different diets, only samples from young SouthCN tigers were selected: *N*(meat) = 5, *N*(milk) = 4, and *N*(mix) = 6. As the effect of age was studied, only samples from meat‐fed SouthCN tigers were selected: *N*(adult) = 7 and *N*(young) = 5.

Similarly, 32 samples were sequenced for fungal community analysis. When the effects of subspecies were studied, samples from adult meat‐fed tigers were selected and grouped into three groups, namely Bengal (*N* = 6), Amur (*N* = 6), and SouthCN tigers (*N* = 7). The effects of different diets were studied by separating the samples into two groups: meat‐fed (*N* = 5) and mix‐fed tigers (*N* = 8). For the milk‐fed group, a total of eight samples from milk‐fed tigers were collected, but seven of them failed to generate an ITS1 amplicon band. When the effect of age was studied, the samples were separated into two groups: adult (*N* = 7) and young tigers (*N* = 5). To study the effect of inheritance, the same four families were studied that had been used for bacterial microbiota analysis.

### 2.3 Data analysis

Sequenced reads were submitted to analysis using the microbial diversity platform of BMKCloud (http://www.biocloud.net/) for quality control, operational taxonomic unit (OTU) assignment, OTU annotation, alpha and beta diversity estimations, principal coordinate analysis (PCoA), unweighted pair group method with arithmetic means (UPGMA) clustering, evaluation of linear discriminatory analysis (LDA) effect size (LEfSe), and function prediction. In brief, sequences with 97% similarity were set as the same OTU using QIIME (Caporaso et al., 2010). OTU abundances were normalized to the sample with the lowest counts. OTUs of 16S rDNA sequences were annotated by alignment with sequences in the SILVA database, whereas OTUs of the ITS1 sequences were annotated by alignment with sequences in the UNITE database with a blast threshold of 0.8 (Nilsson et al., 2018; Quast et al., 2013). Parameters in LEfSe analysis (Segata et al., 2011) were set as log(LDA) >4.0 and *p* < .05. Function prediction of bacteria and fungi was conducted using PICRUSt (http://picrust.github.com/picrust/) and FUNGuild (Nguyen et al., 2016), respectively. For comparisons of alpha diversity, a *t* test was performed to analyze the significance of the differences between groups using the R package. For comparisons of beta diversity, PERMANOVA test was conducted to test the significance of the differences among groups using the R package.

## 3 RESULTS

### 3.1 Bacterial communities in the guts of tigers

#### 3.1.1 Data assessment in 16S rDNA analysis

**Figure A1:**
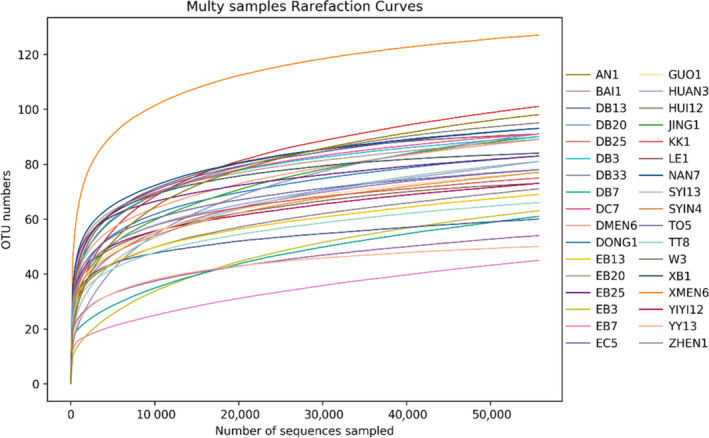
Rarefaction curves of each sample in 16S rDNA analysis

A total of 34 samples were sequenced for analysis of bacterial communities in tiger guts (Table 1). Each sample contained more than 58,697 effective sequences. Rarefaction curves showed that these sequence depths were sufficient for capturing the major bacterial groups in each sample (Figure A1). More than 96% of OTUs could be annotated at the genus level in each sample (Table A1).

#### 3.1.2 General pattern of tiger intestinal bacterial microbiota

**Figure 1:**
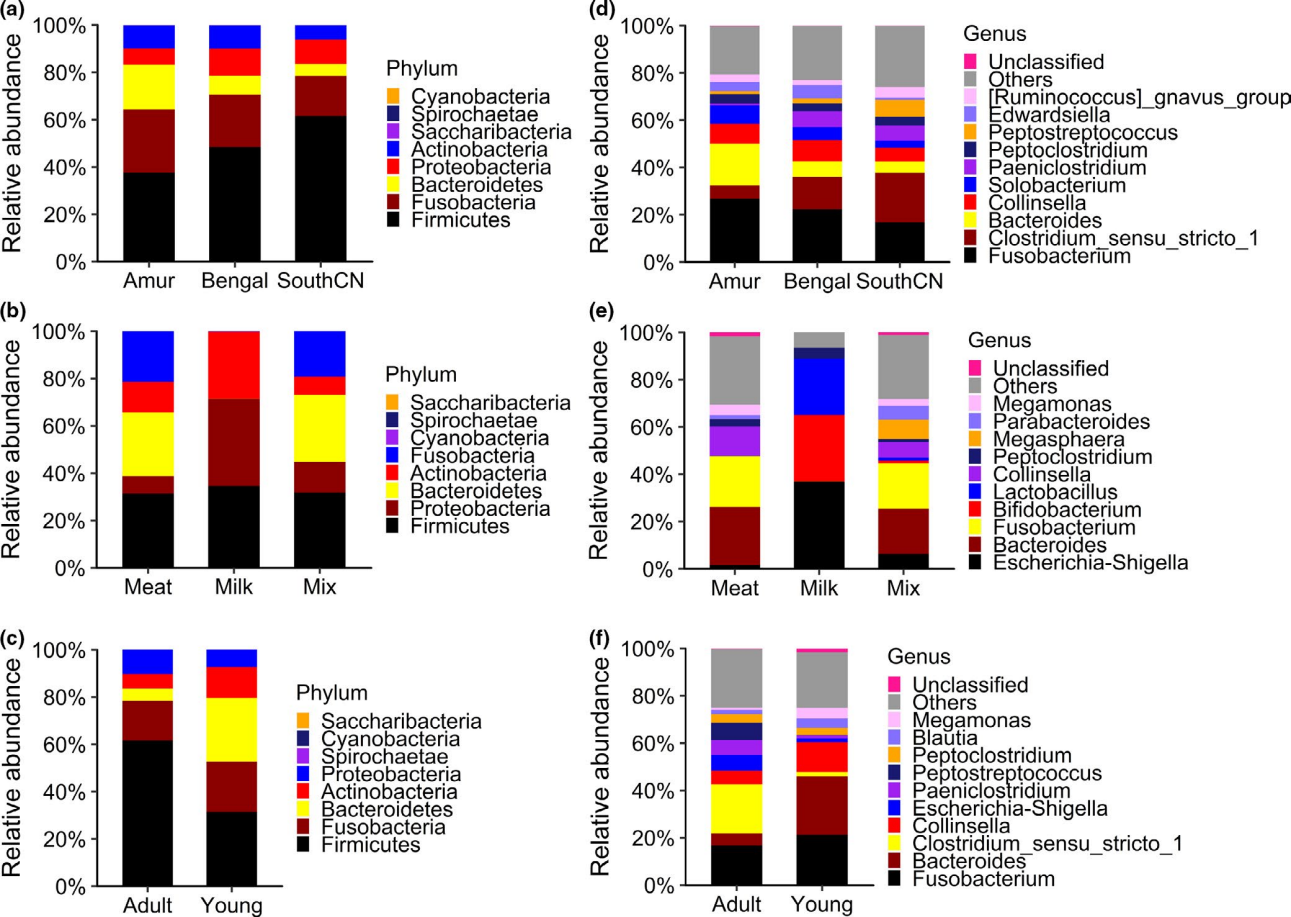
Relative abundances of intestinal bacteria at phylum (a–c) and genus (d–f) levels

According to their relative abundances, bacterial communities in the tiger gut microbiota were similar among subspecies, but differed with diet and age (Figure 1). The composition of the gut bacterial communities of meat‐fed and mix‐fed tigers was similar, but differed from that of milk‐fed tigers. Generally, the bacterial microbiota of the tiger gut showed a predominance of Firmicutes, Fusobacteria, Bacteroidetes, Proteobacteria, and Actinobacteria at the phylum level. In adult tigers fed with meat, the average relative abundances of the dominant bacteria were 49.84% Firmicutes, 21.71% Fusobacteria, 10.33% Bacteroidetes, 9.61% Proteobacteria, and 8.47% Actinobacteria. Other minority phyla were Saccharibacteria, Spirochaetae, and Cyanobacteria, with relative abundances of <0.1% each. At the genus level, the dominant bacteria were *Fusobacterium* (21.71% in adult tigers), *Clostridium *sensu* stricto* 1 (13.84% in adult tigers), *Bacteroides* (9.47% in adult tigers), *Collinsella* (7.60% in adult tigers), *Solobacterium* (5.31% in adult tigers), and *Paeniclostridium* (4.70% in adult tigers).

Unlike the gut microbiota of meat‐fed and mix‐fed tigers, few Fusobacteria and Bacteroidetes colonized the gut of tiger cubs fed with milk. Instead, 99.71% of bacteria in milk‐fed cub guts were Proteobacteria, Firmicutes, and Actinobacteria. At the genus level, the dominant bacteria were *Escherichia/Shigella* (36.88%), *Bifidobacterium* (28.06%), and *Lactobacillus* (23.78%).

#### 3.1.3 Comparison of bacterial diversity indices among groups

**Figure 2:**
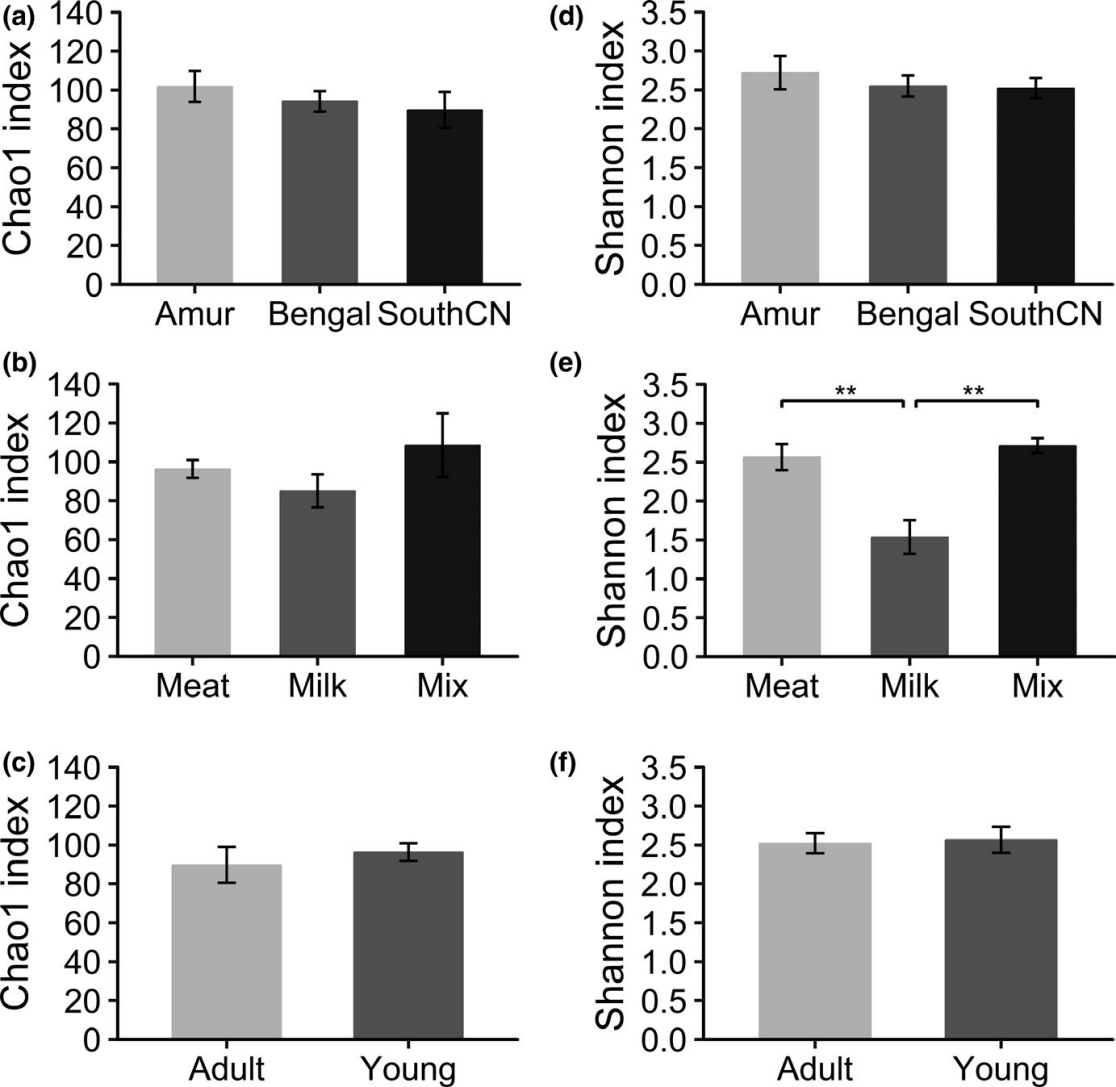
Comparison of Chao1 (a–c) and Shannon (d–f) indices for intestinal bacterial communities categorized by subspecies, diet, and age. ** represents *p* < .01 in statistical tests

In the gut bacterial microbiota, the comparison of Shannon indices showed that milk‐fed tigers had significantly lower community diversity than meat‐fed and mix‐fed tigers, whereas mix‐fed tigers showed no significant differences in community diversity compared with meat‐fed tigers (Figure 2e). These results revealed that the alpha diversity of the gut bacterial community increased when meat was added to the diet. However, no significant differences were observed in alpha diversity between adult and young tigers, or among subspecies (Figure 2d,f). No significant differences were observed in community richness (Chao1 indices) among the subspecies, and between diet or age groups (Figure 2a–c).

**Figure 3:**
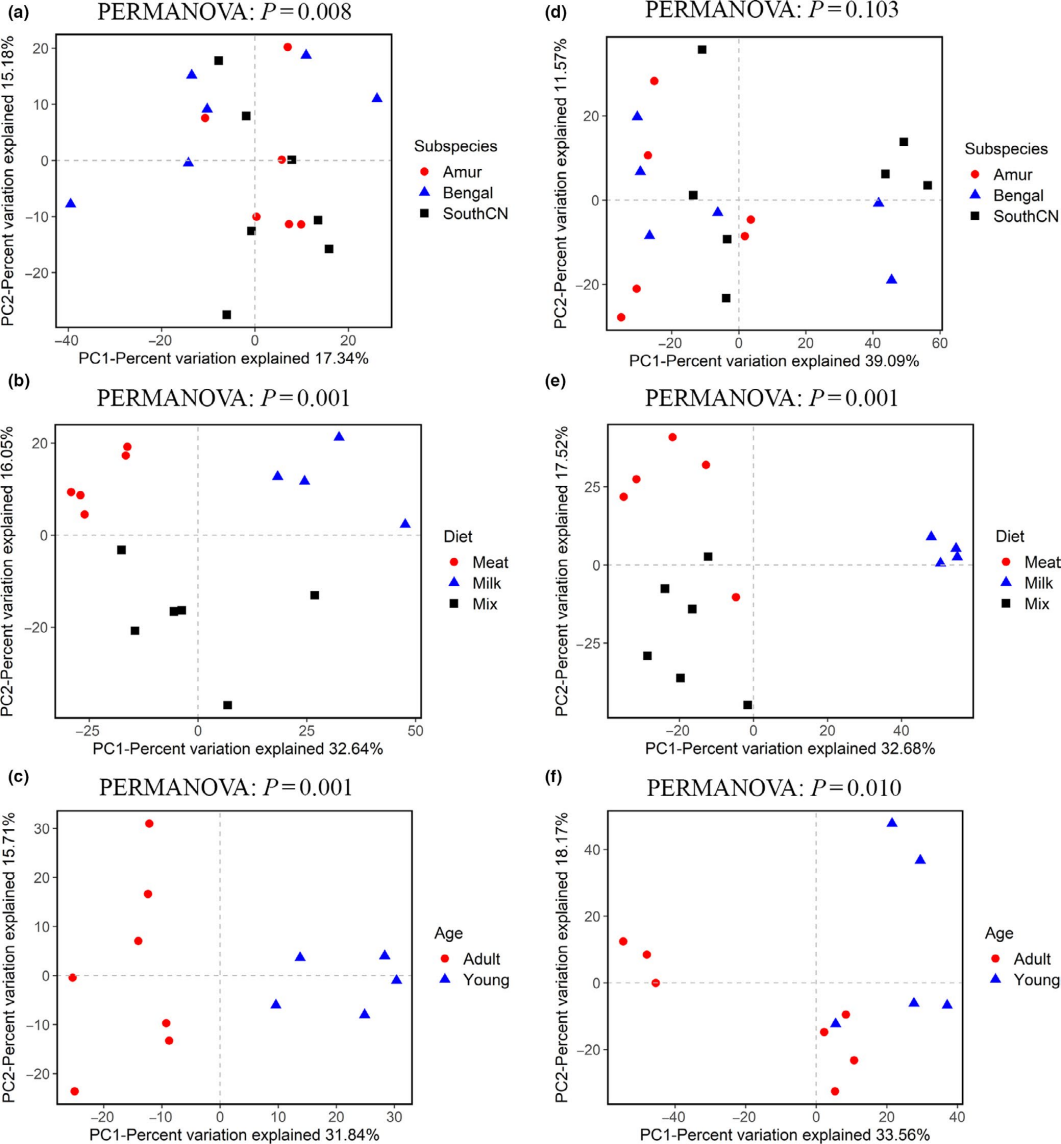
Principal coordinate analyses (PCoA) of the intestinal bacterial microbiota based on binary Jaccard (a–c) and Bray–Curtis (d–f) distance matrices at the operational taxonomic unit (OTU) level. The variation explained by the plotted principal coordinates is indicated in the axis label. *p*‐values of PERMANOVA tests are noted at the top of each PCoA plot

**Figure 4:**
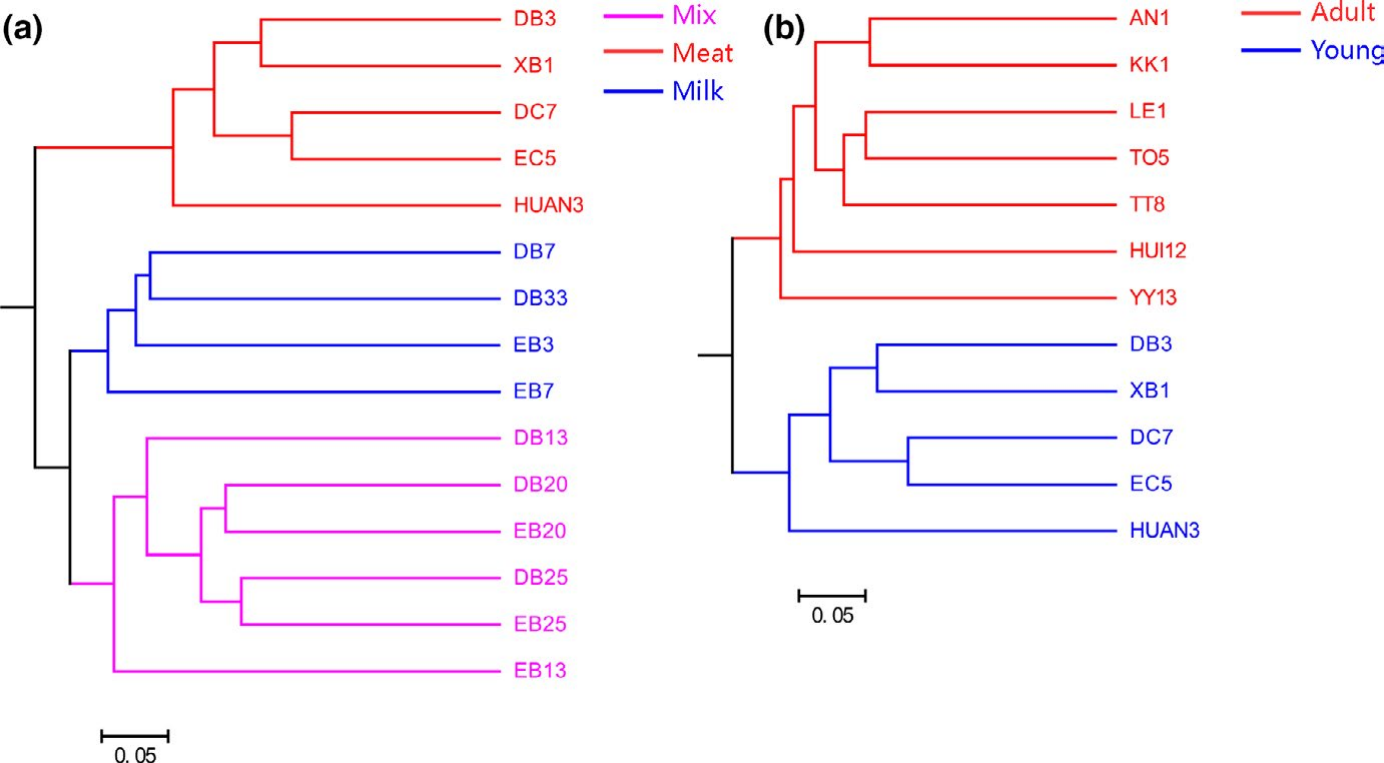
Unweighted pair group method with arithmetic mean (UPGMA) clusters of the intestinal bacterial microbiota based on binary Jaccard distance matrix. Effects of diet (a) and age (b) are shown

**Figure A2:**
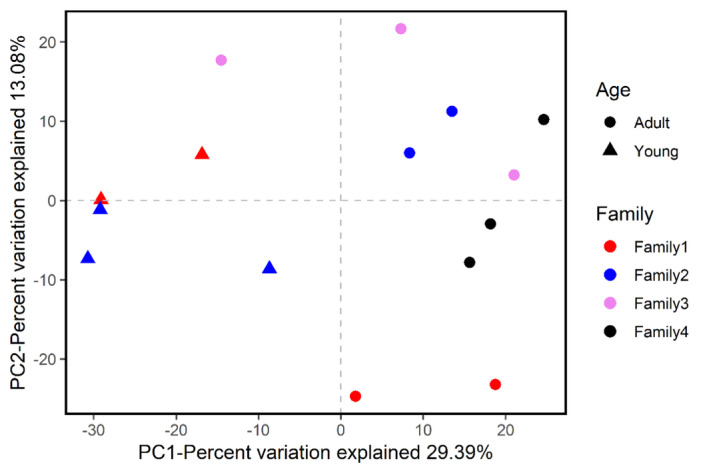
Impact of family on the intestinal bacterial microbiota. The PCoA analysis was conducted based on the binary Jaccard distance matrix at the OTUs level

In terms of beta diversity, according to PCoA analysis, the samples were separated by diet and age, but no clusters by subspecies were observed (Figure 3). These separation patterns were also supported by UPGMA cluster analyses (Figure 4) and PERMANOVA tests (*p* < .05, Figure 3). These results indicated that the beta diversity of gut bacterial microbiota was significantly different depending on diet and age, whereas the differences in beta diversity among subspecies were not significant. In addition, PCoA analysis of four tiger families also showed that these samples were not clustered by family according to the genetic relationship, but by age (Figure A2).

#### 3.1.4 Comparison of bacterial taxa among groups

**Figure 5:**
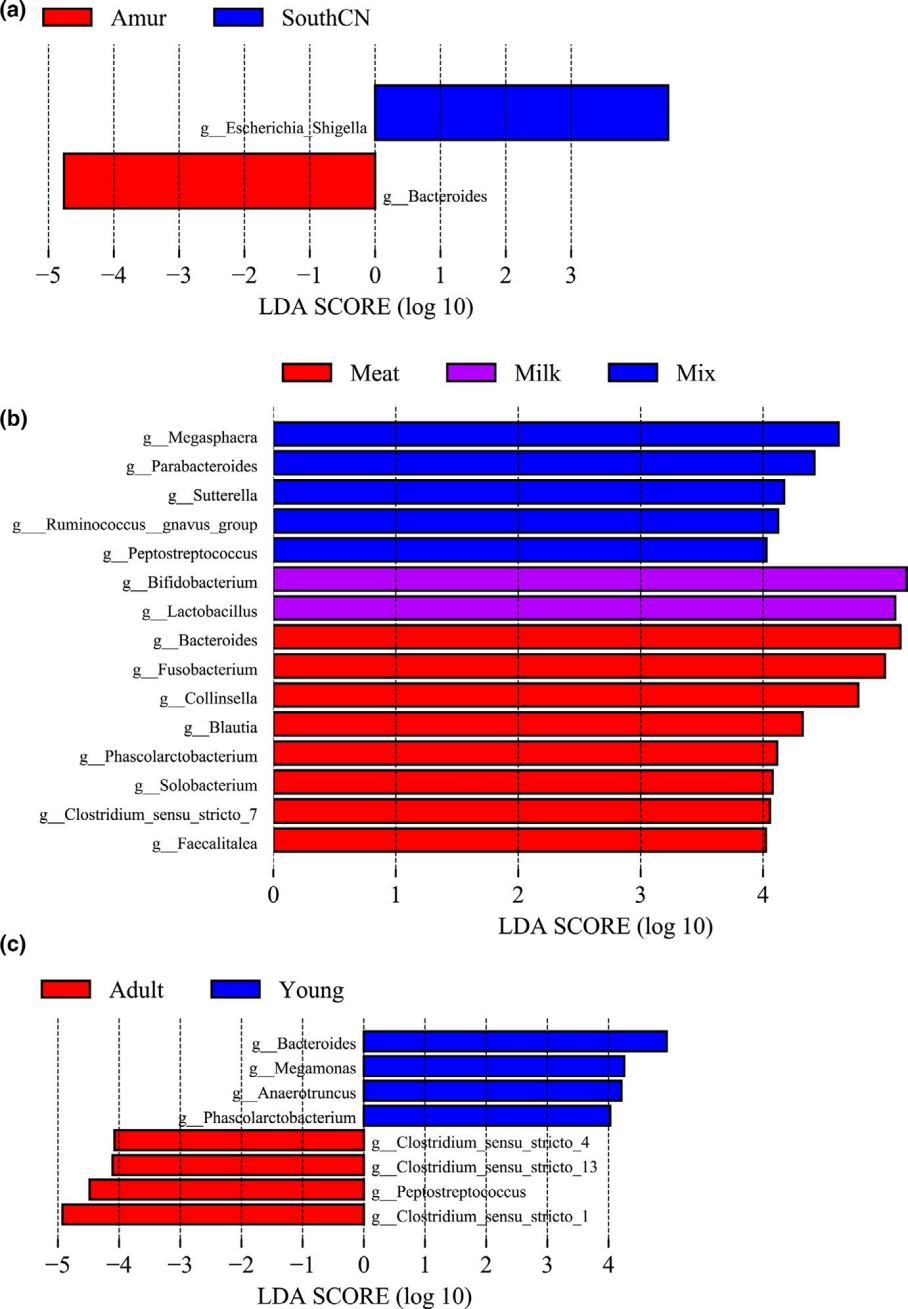
Linear discriminatory analysis (LDA) effect size (LEfSe) determination of the intestinal bacterial microbiota categorized by subspecies (a), diet (b), and age (c)

In the LEfSe analyses, only genus and species levels were selected for use in screening differential bacteria among groups, as more than 96% of OTUs from 16S rDNA sequencing were well annotated at the genus level (Table A1). Figure 5 only shows the results of the LEfSe analysis at the genus level.

Among the three subspecies, SouthCN tigers had a significantly higher abundance of *Escherichia/Shigella*, whereas Amur tigers had a significantly higher abundance of *Bacteroides* (Figure 5a).

When the effects of the different diets on the tiger gut bacterial microbiota were compared, the meat‐fed group showed increased abundances of *Bacteroides, Fusobacterium*, *Collinsella*, *Blautia*, *Phascolarctobacterium*, *Solobacterium*, *Clostridium *sensu* stricto* 7, and *Faecalitalea*. Milk‐fed tigers had significantly higher abundances of *Bifidobacterium* and *Lactobacillus*. However, mix‐fed tigers had higher abundances of *Megasphaera*, *Parabacteroides*, *Sutterella*, *Ruminococcus gnavus* group, and *Peptostreptococcus* in the gut microbiome (Figure 5b).

Compared to adult tigers, young tigers had higher abundances of *Bacteroides*, *Megamonas*, *Anaerotruncus,* and *Phascolarctobacterium*. However, young tigers had lower abundances of *Clostridium *sensu* stricto* 1, *Clostridium *sensu* stricto* 4*, Clostridium *sensu* stricto* 13*,* and *Peptostreptococcus* (Figure 5c).

#### 3.1.5 Function prediction of bacterial communities and comparison between groups

**Figure A3:**
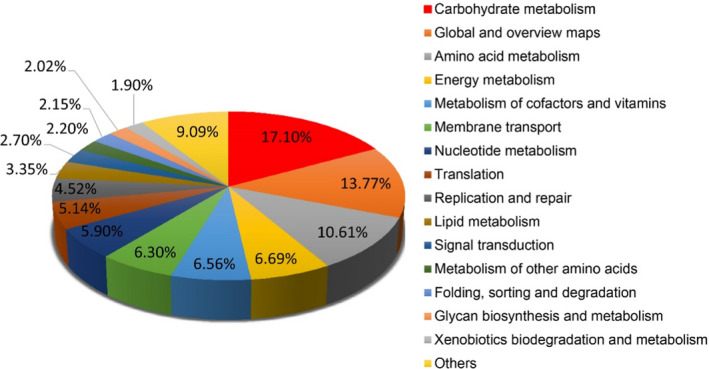
Distribution of functional categories for the intestinal bacterial microbiota. The percentage of each sector was presented by the mean value

Distributions of functional classes were similar among the samples. The main functions of gut bacteria in tigers were carbohydrate metabolism, amino acid metabolism, energy metabolism, and metabolism of cofactors and vitamins, among others, according to the annotation in the KEGG database (Figure A3).

**Figure 6:**
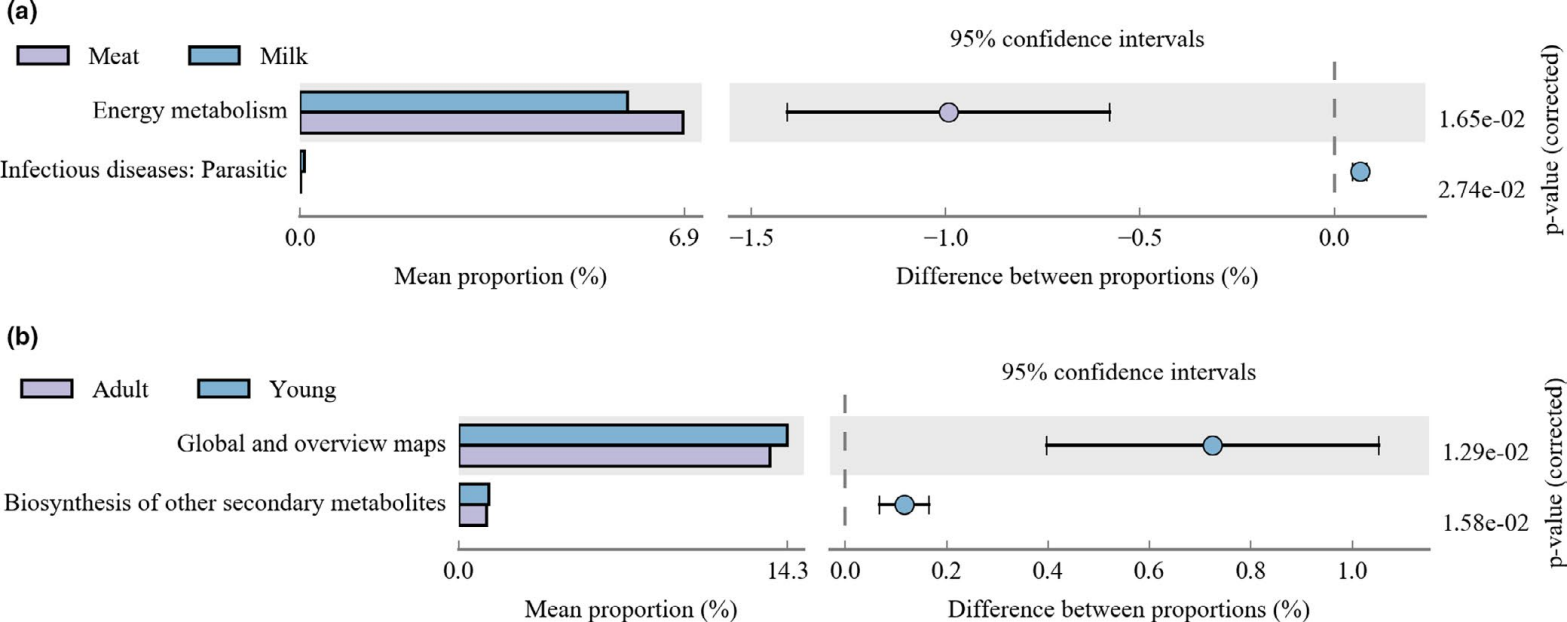
Difference in the function of the intestinal bacterial microbiome regarding diet (a) and age (b)

The comparison of meat‐fed and milk‐fed tigers revealed significant differences in energy metabolism and infectious diseases (parasitic) (Figure 6).

When the effects of age on intestinal microbial functions were analyzed, significant differences were noted in global and overview maps, as well as in the biosynthesis of other secondary metabolites in adult and young tigers (Figure 6).

### 3.2 Fungal communities in the guts of tigers

#### 3.2.1 Data assessment in ITS1 analysis

**Figure A4:**
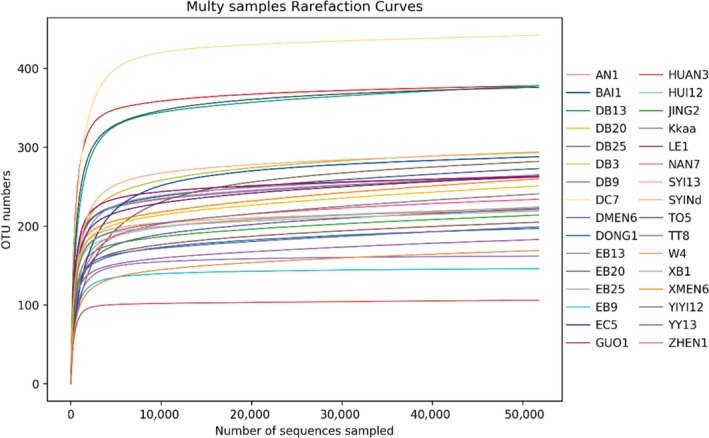
Rarefaction curves of each sample in ITS1 analysis

A total of 32 samples were sequenced for analyzing intestinal fungal composition in tigers (Table 1). Each sample contained at least 57,336 effective sequences. Rarefaction curves showed that these sequence depths were sufficient for capturing the major fungal groups in each sample (Figure A4). However, OTU annotation of ITS1 rDNA was not as detailed as that of 16S rDNA (Table A2).

#### 3.2.2 General pattern of tiger intestinal mycobiota

**Figure 7:**
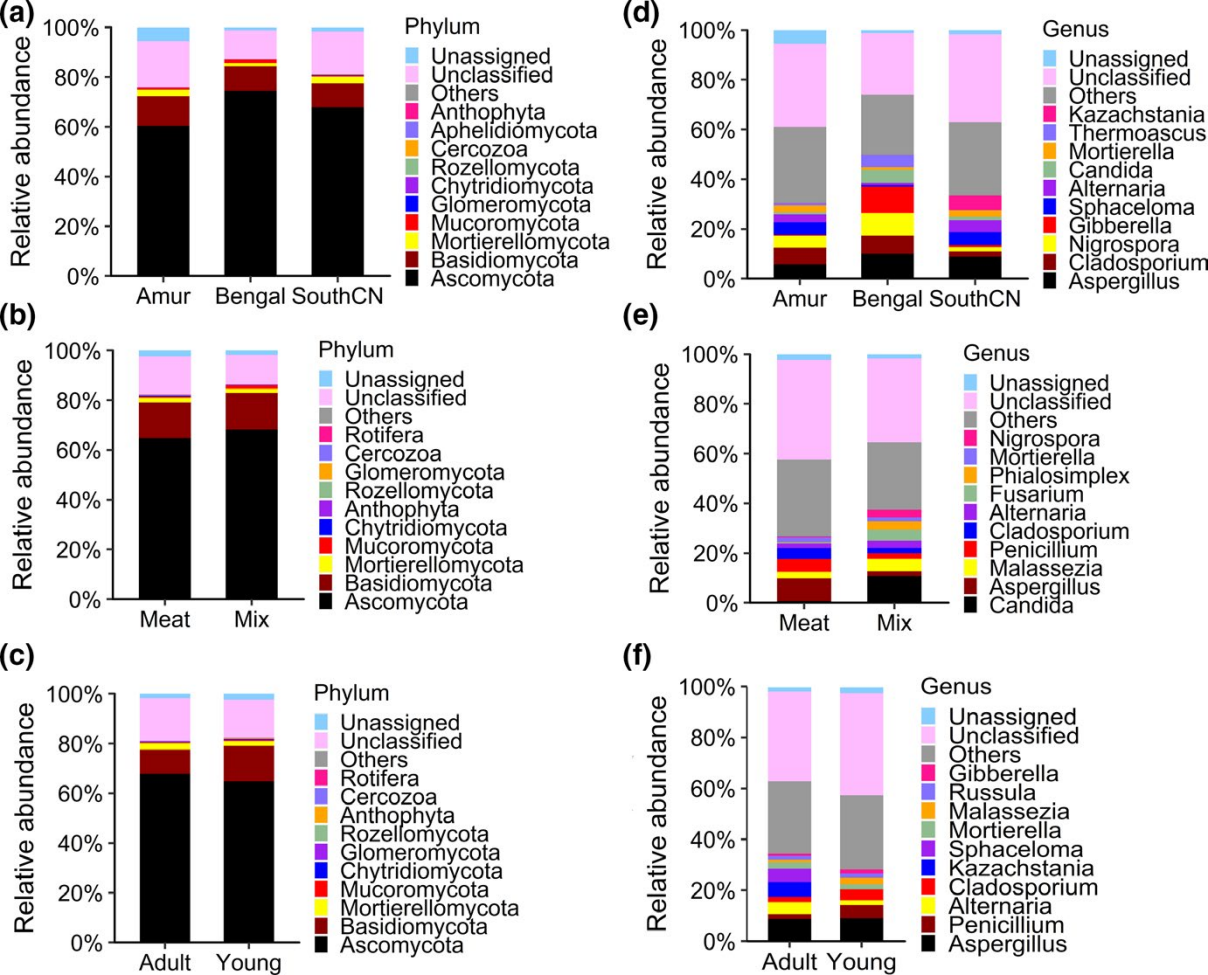
Relative abundances of intestinal fungi at phylum (a–c) and genus (d–f) levels

The composition of the fungal community in the gut of tigers was similar among subspecies, as well as between different diet, and age groups (Figure 7). In sum, the tiger intestinal fungi belonged to 11 phyla, 31 classes, 69 orders, 171 families, 304 genera, and 344 species.

The gut mycobiota of tigers was dominated by Ascomycota and Basidiomycota, which accounted for 78.04% of the average abundance in adult tigers (Figure 7). The dominant genera of tiger intestinal fungi were *Aspergillus* (8.28%), *Cladosporium* (5.19%), *Nigrospora* (5.04%), *Gibberella* (3.75%), *Sphaceloma* (3.69%), and *Alternaria* (3.04%).

#### 3.2.3 Comparison of fungal diversity indices among groups

**Figure 8:**
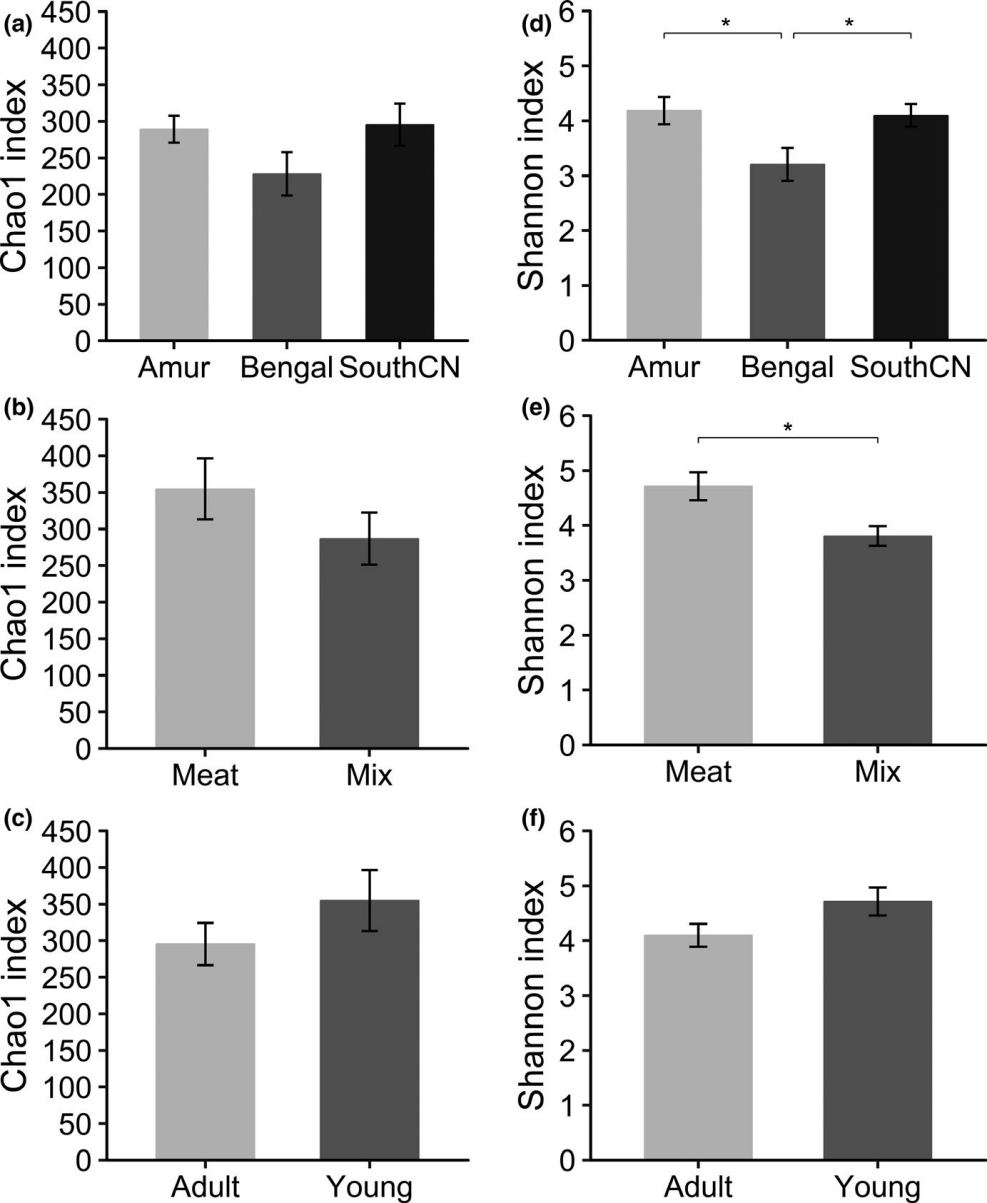
Comparison of Chao1 (a–c) and Shannon (d–f) indices for the intestinal fungal communities categorized by subspecies, diet, and age. * represents *p* < .05 in statistical tests

For alpha diversity, no significant differences existed in community richness (Chao1 indices) among subspecies, diet, and age groups (Figure 8a–c). Comparison of Shannon indices revealed that community diversity of the gut mycobiota was significantly lower in Bengal than Amur or SouthCN tigers (Figure 8d). Meat‐fed tigers were characterized by significantly higher community diversity than mix‐fed tigers (Figure 8e). However, there were no significant differences in alpha diversity between age groups (Figure 8f).

**Figure 9:**
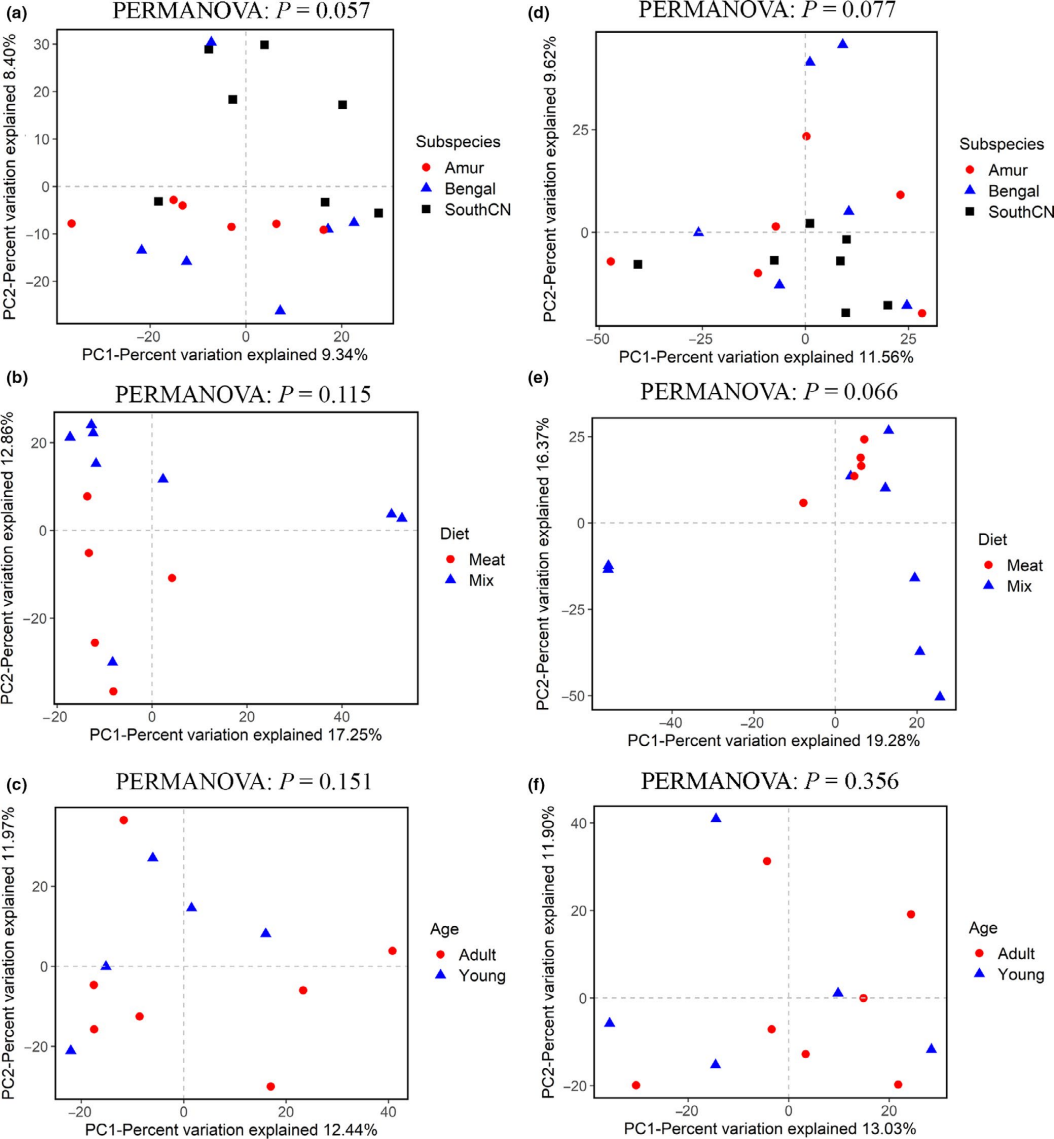
Principal coordinate analyses (PCoA) of the intestinal mycobiota based on binary Jaccard (a–c) and Bray–Curtis (d–f) distance matrices at the operational taxonomic unit (OTU) level. The variation explained by the plotted principal coordinates is indicated in the axis label. *p‐*values of PERMANOVA tests are noted at the top of each PCoA plot

For beta diversity, the tigers’ intestinal fungal samples could not be separated by subspecies, diet, or age according to PCoA (Figure 9) and UPGMA cluster analyses. PERMANOVA tests also showed no significant differences in beta diversity among subspecies, diet, or age groups (*p* > .05, Figure 9).

#### 3.2.4 Comparison of fungal taxa among groups

**Figure 10:**
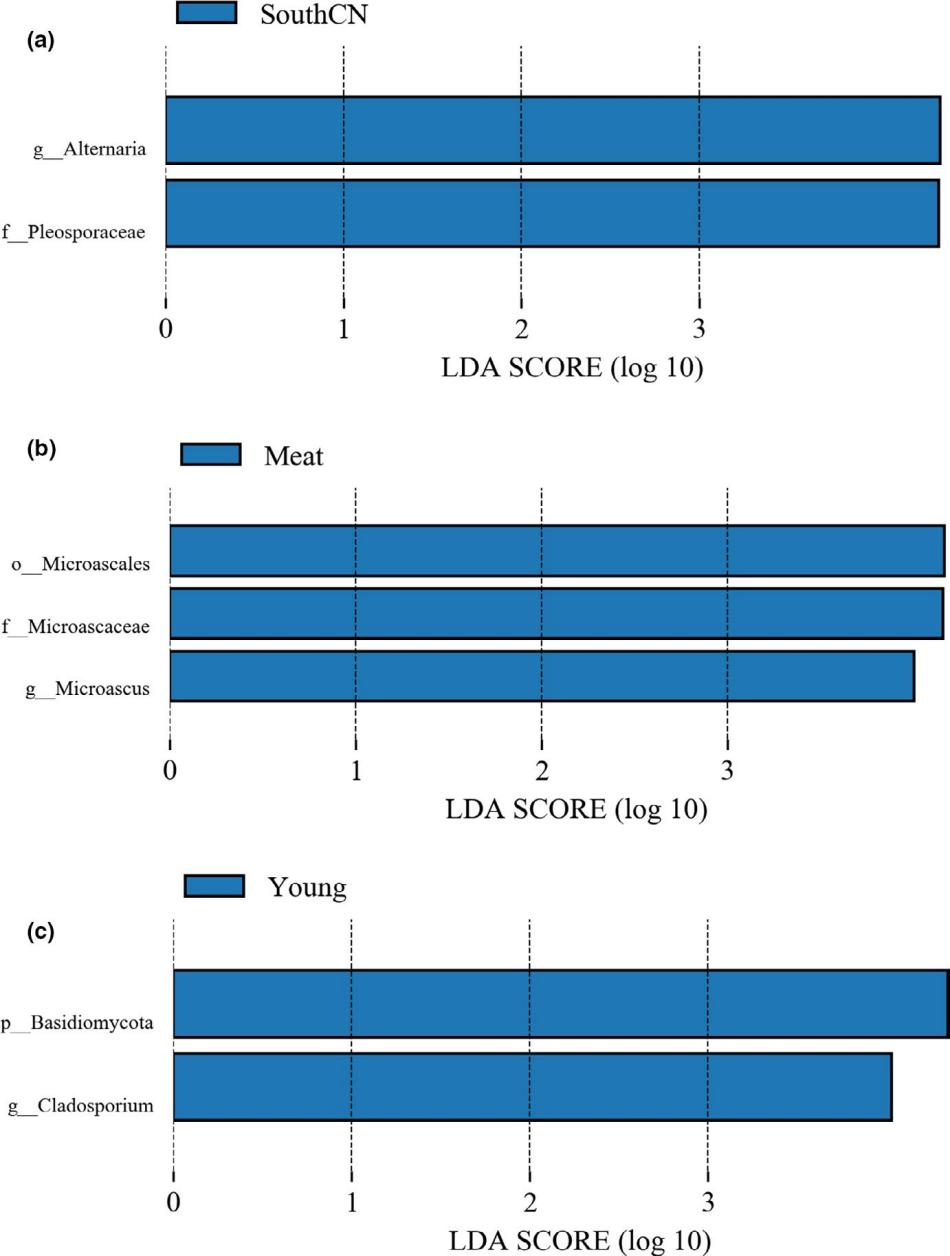
Linear discriminatory analysis (LDA) effect size (LEfSe) of the intestinal mycobiota regarding subspecies (a), diet (b), and age (c) categories

Among the three subspecies studied, the SouthCN tigers showed significantly higher abundances of *Alternaria* and Pleosporaceae (Figure 10a). Regarding dietary effects on the tiger intestinal fungal community, abundances of Microascales, Microascaceae, and *Microascus* were significantly increased in the guts of meat‐fed tigers compared with that of mix‐fed tigers (Figure 10b)*.* Compared to adult tigers, young tigers were characterized by significantly higher abundances of Basidiomycota and *Cladosporium* in the gut mycobiota (Figure 10c)*.*

#### 3.2.5 Function prediction of fungal communities and comparison between groups

**Figure 11:**
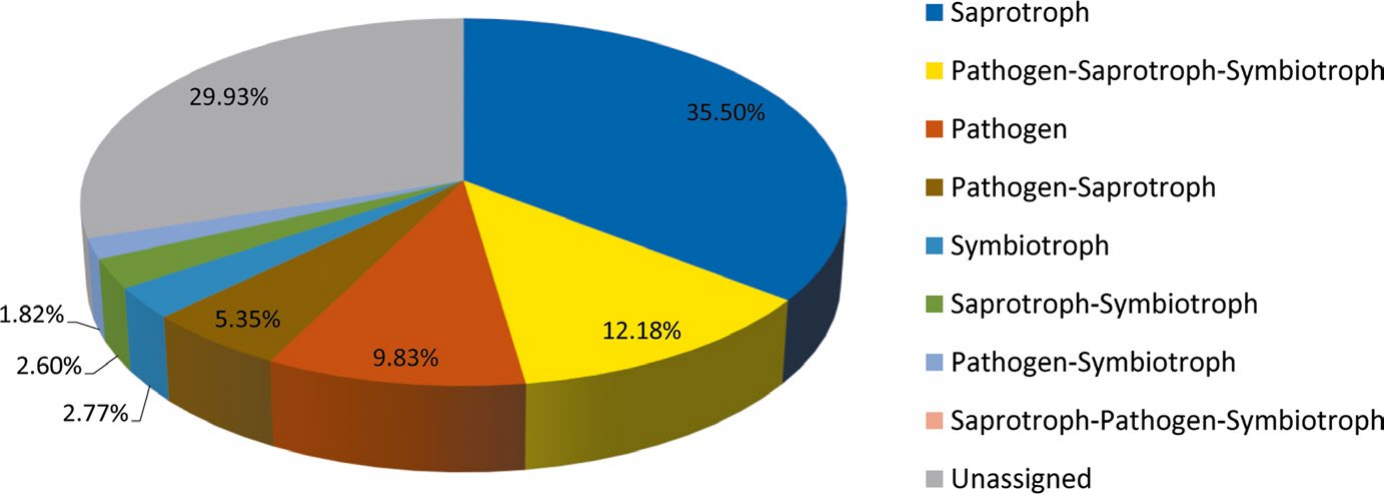
Distribution of functional categories of the gut mycobiota. The mean percentages of each sector are presented

The function of tiger intestinal fungi was categorized into eight trophic modes. Among these trophic modes, saprotrophs were the most abundant fungal guild. Notably, 9.83% of fungal sequences, on average, were predicted to be derived from pathogenic fungi (Figure 11). Among the predicted pathogens, 13 genera were predicted to be animal pathogens or fungal parasites: *Cutaneotrichosporon, Cystobasidium, Engyodontium, Hirsutella, Lecanicillium, Metacordyceps, Metarhizium, Microsporum, Pochonia, Purpureocillium, Septobasidium, Simplicillium,* and *Trichosporon.* Among these genera, *Trichosporon* was present in 23 of the 32 analyzed samples. The other genera were only present in 1–15 samples (Table A3).

## 4 DISCUSSION

Understanding the factors that cause changes in the composition and function of the gut microbiota could provide insights into possible measures to promote the health of endangered animals by targeting these microbial communities in clinical treatment and health management settings. Using high‐throughput sequencing technology, the characteristics of the tiger intestinal microbiota and the factors underlying the observed differences were comprehensively investigated in this study.

The gut bacterial microbiota in tigers is generally dominated by Firmicutes, Fusobacteria, Bacteroidetes, Proteobacteria, and Actinobacteria. This finding is consistent with previous studies on tiger intestinal bacterial composition (Peng, 2017; Tian, 2013). The pattern of the bacterial microbiota in the tiger gut is similar to that in other mammals, except for the significantly higher abundance of Fusobacteria (21.71% in adult tigers) (Ley et al., 2008). Fusobacteria were all contributed by *Fusobacterium*. A higher abundance of *Fusobacterium* has been associated with a series of diseases in humans, such as colorectal carcinoma and acute appendicitis (Brennan & Garrett, 2016; Zhong, Browersinning, Firek, & Morowitz, 2014). In addition to *Fusobacterium*, some species of *Clostridium,* another dominant genus, have been reported to be associated with colorectal carcinoma in humans. *Clostridium perfringens* type A was also reported to be associated with severe hemorrhagic enterocolitis in tigers (Zhang, Hou, & Ma, 2012). However, the rate of colorectal carcinoma in tigers is far below that observed in humans (Tian, 2013). According to previous studies, hosts with a high abundance of Fusobacteria in the gut are usually predators, such as seals, dolphins, black jackals, dogs, cheetahs, polar bears, fringe‐lipped bats, vultures, and alligators (Keenan, Engel, & Elsey, 2013; Ley et al., 2008; Nelson, Rogers, Carlini, & Brown, 2013; Nishida & Ochman, 2017; Roggenbuck et al., 2014). Moreover, meat‐fed tigers showed increased abundances of *Fusobacterium* compared with mix‐fed and milk‐fed tigers. Together, these results support the assumption that the high abundance of *Fusobacterium* was associated with the digestion of meat. The capacity of degrading proteins has been found in *Fusobacterium nucleatum,* which contains a 65 kDa protease that can degrade fibrinogen, fibronectin, collagen I, and collagen IV (Bachrach, Rosen, Bellalou, Naor, & Sela, 2004). Correspondingly, according to the predicted functional profiles, the intestinal bacteria in meat‐fed tigers contributed to the significant increase in energy metabolism pathways compared with milk‐fed tigers, which implied that the gut of meat‐fed tigers had more bacteria to assist with energy metabolism.

The gut microbiota of endangered animal offspring has rarely been characterized. In this study, the effects of different diets on the gut microbiota of young tigers under 6 months of age were investigated. A comparison of alpha and beta diversity indices among groups showed that the diversity of bacterial microbiota in the gut of milk‐fed tigers was significantly different from that in the mix‐fed and meat‐fed tigers’ gut. This was consistent with previous studies in other vertebrates, implying that the diet could shape the gut microbiota (Ley et al., 2008; Nishida & Ochman, 2017). The intestinal bacterial microbiota in milk‐fed cubs was characterized by low diversity and was dominated by *Escherichia/Shigella*, *Bifidobacterium,* and *Lactobacillus.* This finding was similar to observations made in formula‐fed human infants (Azad et al., 2013; Penders et al., 2006) and corresponded to the milk‐fed tiger diet, which included some probiotics. Milk‐fed tigers showed significantly higher abundances of *Bifidobacterium* and *Lactobacillus*, and a lower abundance of *Fusobacterium*. *Bifidobacterium* and *Lactobacillus* species are well known for their role in milk fermentation and are hence widely used as probiotics (Widyastuti & Febrisiantosa, 2014; Xiao et al., 2003). In addition, bifidobacterial diversity is believed to play a role in the development of the host immune system in early life (Sjogren et al., 2009). Although the periods of diet change were short, young tigers with a change in diet from milk to meat displayed increased alpha diversity of the intestinal bacterial microbiota, changes in dominant groups of bacteria, and decreased levels of bacteria that could improve milk digestion, such as *Bifidobacterium* and *Lactobacillus* (Jiang, Mustapha, & Savaiano, 1996; Widyastuti & Febrisiantosa, 2014; Xiao et al., 2003). In contrast, bacteria that are associated with meat digestion gradually increased, such as *Fusobacterium* and *Bacteroides* (Bachrach et al., 2004; Davila et al., 2013; Macfarlane, Allison, Gibson, & Cummings, 1988; Riepe, Goldstein, & Alpers, 1980). Differences in intestinal bacterial function showed that milk‐fed tigers had significantly lower abundances of bacteria associated with energy metabolism and higher abundances of pathogenic bacteria. This result implied that the diet not only changed the metabolism, but might also be important for pathogen invasion. For example, guts of milk‐fed tigers were enriched for *Escherichia*/*Shigella*, which were significantly associated with diarrheal disease in infants (Pop et al., 2014). In addition to milk‐fed cubs, meat‐fed cubs also showed a significant difference in beta diversity of their gut bacterial microbiota compared with adult tigers. This was consistent with previous results showing that the composition of the gut microbiota varies with age (Rothschild et al., 2018). Age‐related shifts in bacterial abundance in tigers included *Bacteroides*, *Megamonas*, *Anaerotruncus*, *Phascolarctobacterium, Peptostreptococcus,* and *Clostridium* groups. Among these genera, changes with age in *Bacteroides* and *Clostridium* groups have also been observed in humans (Clark & Walker, 2018). The different intestinal bacterial microbiota between adult and young tigers is explained by the metabolic function of global and overview maps and biosynthesis of other secondary metabolites (Figure 6), which are consistent with active metabolism in young tigers.

Previous studies of the mammalian gut microbiota showed that host taxonomy structured the intestinal bacterial microbiota at the order, family, and genus levels; the fecal microbial communities in the same species were more similar to each other than to communities of different species (Ley et al., 2008; Nishida & Ochman, 2017). However, at the intraspecies level, the diversity of the bacterial microbiota in the tiger gut was not significantly changed by the tiger subspecies. Moreover, when the effect of heredity is considered, the gut microbiota clustered by age instead of family. These results implied that the bacterial microbiota was less influenced by host intraspecies genetic variation.

Although the gut mycobiome is receiving increased research attention for its potential role in the etiology of gut‐associated diseases, research on the gut mycobiome is still in its infancy (Cotter et al., 2017). Current knowledge on fungal diversity in the gut of vertebrates is still limited to a small number of species, such as humans, mice, dogs, bats, and several fishes (Cotter et al., 2017; Foster, Dowd, Stephenson, Steiner, & Suchodolski, 2013; Li et al., 2018; Siriyappagouder et al., 2018). At the phylum level, tiger intestinal fungal composition was similar to that observed in human, mouse, dog, bat, and fish guts, which are dominated by Ascomycota and Basidiomycota (Cotter et al., 2017; Foster et al., 2013; Li et al., 2018; Siriyappagouder et al., 2018). Like bacterial communities, the gut mycobiota differed among vertebrate species. Dominant genera of the tiger mycobiota were *Aspergillus*, *Cladosporium*, *Nigrospora*, *Gibberella*, *Sphaceloma*, and *Alternaria. Aspergillus* and *Cladosporium* were also found dominant in the human gastrointestinal tract (Lai, Tan, & Pavelka, 2019), but the other dominant genera were different between tigers and other vertebrates. For example, *Candida* was the most abundant genus found in dogs, humans, and phytophagous bats (Foster et al., 2013; Lai et al., 2019), but it was not found in the tiger gut. This difference may be associated with the high protein content in the tiger diet, because *Candida* was negatively correlated with diets high in amino acids, proteins, and fatty acids, but was positively correlated with diets high in carbohydrates (Hoffmann et al., 2013).

Similar to bacterial communities, beta diversity of the fungal microbiota was not significantly influenced at the subspecies level. However, alpha diversity of the fungal microbiota was significantly different among the subspecies analyzed. Previous studies have shown that changes in diet can regulate mycobiota diversity in humans and bats (Hallen‐Adams & Suhr, 2016; Li et al., 2018). In contrast, no significant differences in intestinal fungal beta diversity were found between meat‐fed and mix‐fed tigers. Remarkable similarities in intestinal fungal diversity between diets were observed in laboratory‐reared zebrafish (Siriyappagouder et al., 2018). However, the similarity of intestinal fungal beta diversity in tigers might have resulted from the similarity of diets between meat and mix groups. Significant differences in intestinal fungal alpha diversity were found. In addition, for seven out of eight samples from milk‐fed tigers, we failed to amplify ITS1 sequences, which implied that milk‐fed tigers had fewer intestinal fungi than meat‐fed or mix‐fed tigers. Regarding the effect of age, which is inconsistent with research on the human gut mycobiota, which has shown significant differences with respect to age and gender (Strati et al., 2016), the tiger gut mycobiota was not significantly influenced by age. In contrast to the intestinal bacterial community of universal character, changes in the intestinal fungal community may vary with species. Future research needs to further explore the variation in gut mycobiota in other animals.

More than one‐third of the fungal species present in the tiger gut were saprotrophic (Figure 11). Saprotrophic fungi decompose and redistribute nutrients (Hättenschwiler, Tiunov, & Scheu, 2005) and could therefore provide benefits to a growing host. In addition, commensal fungi in the gut may have potential health benefits or probiotic effects, such as those provided by yeasts (Hatoum, Labrie, & Fliss, 2012). Although all tigers included were apparently healthy, approximately 9.83% of intestinal fungi were categorized as pathogenic guilds. These fungi may serve as opportunistic pathogens, especially the genera that were cataloged into animal pathogens and detected in only a few samples. For example, *Purpureocillium lilacinum* has been reported to cause infections in both immunocompromised and immunocompetent humans (Saghrouni et al., 2013). *Engyodontium album* causes diseases in bovines and humans (Balasingham et al., 2011; Thamke, Mendiratta, Dhabarde, & Shukla, 2015). Moreover, a high frequency of azole resistance has been found in fungal isolates from human guts (Strati et al., 2016). The intestine appears to serve as a reservoir of opportunistic pathogens in plants and animals. The role and pathogenic potential of intestinal fungi need further investigation.

## 5 CONCLUSION

Overall, this study provides comprehensive information on the gut microbiota of tigers and further describes the different impacts of host intraspecies genetic variation, diet, and age on gut bacterial and fungal communities.

The impacts of subspecies, diet, and age on intestinal bacterial and fungal communities were different. The bacterial community in the tiger gut was dominated by Firmicutes, Fusobacteria, Bacteroidetes, Proteobacteria, and Actinobacteria. The bacterial microbiota was significantly changed by differences in diet and age, whereas host genetic variation within species had no significant impact on the gut bacterial microbiota. Meat‐fed tigers had significantly higher abundances of *Bacteroides, Fusobacterium*, *Collinsella*, *Blautia*, *Phascolarctobacterium*, *Solobacterium*, *Clostridium *sensu* stricto* 7, and *Faecalitalea*. Milk‐fed tigers had significantly higher abundances of *Bifidobacterium* and *Lactobacillus*, while tiger cubs showed higher abundances of *Bacteroides*, *Megamonas*, *Anaerotruncus,* and *Phascolarctobacterium*. For the gut mycobiota, the dominant genera were *Aspergillus*, *Cladosporium*, *Nigrospora*, *Gibberella*, *Sphaceloma,* and *Alternaria*. The gut mycobiota in tigers showed no significant differences in beta diversity among subspecies and diet or age groups. However, significant differences were observed in alpha diversity of the gut mycobiota among different subspecies and diets.

Our findings expand the current understanding of the gut microbiota in tigers and even in vertebrates, and these findings provide a reference for the healthy management of tigers by modulating the gut microbiota. Moreover, this baseline information will be crucial for future studies on wildlife mycobiome.

## CONFLICT OF INTERESTS

None declared.

## AUTHOR CONTRIBUTIONS

HJ: conceptualization (equal); formal analysis (lead); funding acquisition (equal); writing‐original draft (lead); writing‐review and editing (equal); WC: resources (equal); LS: resources (equal); MH: formal analysis (supporting); LL: formal analysis (supporting); QS: resources (equal); GL: formal analysis (supporting); HIA: writing‐review and editing (equal); LL: investigation (supporting); XZ: investigation (supporting); HL: investigation (supporting); JC: conceptualization (equal); funding acquisition (equal); resources (equal); supervision (lead); writing‐review and editing (equal).

## ETHICS STATEMENT

This study was carried out following the recommendations of the animal experimental inspection form of the Guangdong Institute of Applied Biological Resources (permit number: GIABR20171101). The protocol was approved by the Committee on the Ethics of Animal Experiments of the Guangdong Institute of Applied Biological Resources.

## Data Availability

All raw sequences are available at the NCBI Sequence Read Archive (SRA) database under accession numbers SRP187406 and SRP187945. Corresponding BioProject numbers are PRJNA525192: https://www.ncbi.nlm.nih.gov/search/all/?term=PRJNA525192 and PRJNA526087: https://www.ncbi.nlm.nih.gov/search/all/?term=PRJNA526087.

## APPENDIX 1

**Table A1:** Percentage of the annotated bacteria at different taxonomical levels in 16S rDNA analysis

Sample	Phylum	Class	Order	Family	Genus	Species
AN1	100	100	100	100	100	64
BAI1	100	100	100	100	100	37
DB13	100	100	100	100	100	99
DB20	100	100	100	100	100	95
DB25	100	100	100	100	99	99
DB3	100	100	100	100	98	89
DB33	100	100	100	100	100	94
DB7	100	100	100	100	100	78
DC7	100	100	100	100	99	99
DMEN6	100	100	100	100	100	87
DONG1	100	100	100	100	100	95
EB13	100	100	100	100	100	94
EB20	100	100	100	100	98	97
EB25	100	100	100	100	96	95
EB3	100	100	100	100	100	92
EB7	100	100	100	100	100	94
EC5	100	100	100	100	99	97
GUO1	100	100	100	100	100	91
HUAN3	100	100	100	100	100	68
HUI12	100	100	100	100	100	97
JING1	100	100	100	100	100	95
KK1	100	100	100	100	100	64
LE1	100	100	100	100	100	97
NAN7	100	100	100	100	100	87
SYI13	100	100	100	100	100	92
SYIN4	100	100	100	100	99	96
TO5	100	100	100	100	100	88
TT8	100	100	100	100	100	77
W3	100	100	100	100	100	75
XB1	100	100	100	100	96	95
XMEN6	100	100	100	100	100	79
YIYI12	100	100	100	100	100	94
YY13	100	100	100	100	100	87
ZHEN1	100	100	100	100	100	94

**Table A2:** Percentage of the annotated bacteria at different taxonomical levels in ITS1 analysis

Sample	Phylum	Class	Order	Family	Genus	Species
AN1	90	86	85	81	76	61
BAI1	90	88	87	78	75	60
DB3	95	90	89	84	76	50
DB9	98	96	96	88	78	43
DB13	87	80	79	72	61	48
DB20	80	77	75	70	56	35
DB25	69	67	66	64	50	35
DC7	68	54	52	44	35	22
DMEN6	94	91	90	86	78	59
DONG1	96	95	94	86	82	50
EB9	98	96	95	90	81	47
EB13	92	91	90	88	86	70
EB20	97	92	92	90	86	48
EB25	80	30	30	29	26	16
EC5	89	86	84	80	74	42
GUO1	82	75	74	67	60	43
HUAN3	82	78	75	68	58	31
HUI12	91	87	86	75	73	42
JING2	58	57	57	49	47	35
Kkaa	72	69	67	63	46	27
LE1	85	83	80	76	65	46
NAN7	93	92	91	73	71	47
SYI13	66	65	65	63	59	39
SYINd	73	70	69	67	56	38
TO5	88	84	82	73	62	32
TT8	62	53	52	49	47	21
W4	95	95	94	92	87	81
XB1	87	83	61	55	50	37
XMEN6	79	72	70	65	59	33
YIYI12	89	86	86	83	77	35
YY13	90	87	86	82	79	22
ZHEN1	97	96	95	93	87	77

**Table A3:** Relative abundances of the fungal genera that were categorized into animal pathogens or fungal parasites

Genus	AN1	BAI1	DMEN6	DONG1	GUO1	HUI12	JING2	Kkaa	LE1
*Cutaneotrichosporon*	0.06%	0	0	0	0.13%	0	0	0	0
*Cystobasidium*	0	0.07%	0	1.75%	0	0	0	0	0
*Engyodontium*	0	0.14%	0.21%	0	0	8.49%	0	0	0.28%
*Hirsutella*	0	0	0	0	0.17%	0	0	0	0
*Lecanicillium*	0	0	0	0	0.11%	0	0	0	0
*Metacordyceps*	0	0	0	0	0	0	0	0.15%	0
*Metarhizium*	<0.01%	0	<0.01%	0	0	0	0	0	0
*Microsporum*	0	0	0	0	0	0	0	0	0
*Pochonia*	0.19%	0	0	0	0	0	0	0	0
*Purpureocillium*	0	0	0	0	0	0	0	0.48%	0
*Septobasidium*	0	0	0	0	0	0	0	0	0
*Simplicillium*	0	0	0	0	0	0	0	0	0
*Trichosporon*	0.09%	0.06%	0.99%	9.02%	0.28%	0	1.26%	0	0.41%

Genus	NAN7	SYI13	SYINd	TO5	TT8	W4	XMEN6	YIYI12
*Cutaneotrichosporon*	0	0	0	0.34%	0	0	0	0
*Cystobasidium*	0	0	0	0.38%	0	0	0	0
*Engyodontium*	<0.01%	0.09%	0	0.07%	0	<0.01%	0	0
*Hirsutella*	0	0	0	0	0	0	0	0
*Lecanicillium*	0.14%	0.22%	0	<0.01%	0	0.08%	0	0
*Metacordyceps*	0	0	0	0	0	0.14%	0	0
*Metarhizium*	0	0	0	0.04%	0	0	0	0
*Microsporum*	0	0	0	0	0	0	0	0
*Pochonia*	0	0	0	0	0	0	0	0
*Purpureocillium*	0	0.03%	0.42%	0	0.48%	0	0	0
*Septobasidium*	0	0	0	0	0	0	0	0
*Simplicillium*	0	0	0	0	0	0	0	0
*Trichosporon*	2.40%	0.05%	0	1.42%	0.05%	0	0.62%	0.18%

Genus	YY13	ZHEN1	DB3	DB9	DB13	DB20	DB25
*Cutaneotrichosporon*	0	0	0	0	0	0	0
*Cystobasidium*	0	0	0.18%	0.03%	0.03%	0	0
*Engyodontium*	0	0	0.16%	9.11%	0.20%	1.53E‐05	0.25%
*Hirsutella*	0	0	0	0	0	0	0
*Lecanicillium*	0	0	0	0.55%	0.13%	0	0
*Metacordyceps*	0.21%	0	0	0	0	0	0
*Metarhizium*	0.20%	0	0	<0.01%	0	0.22%	0.23%
*Microsporum*	0	0	0	0	0	0	0
*Pochonia*	0	0	0	0	0	0	0
*Purpureocillium*	0	0	0.06%	0	0	0	0
*Septobasidium*	0	0	0	0	0	0	0
*Simplicillium*	0	0	0	2.89%	0	0	0
*Trichosporon*	0.55%	0	0.22%	0	0.30%	0.23%	0.21%

Genus	DC7	EB9	EB13	EB20	EB25	EC5	HUAN3	XB1
*Cutaneotrichosporon*	0	0	0	0.26%	0	0	0.21%	0
*Cystobasidium*	0	0	0	0.07%	0	0	0	0
*Engyodontium*	0.16%	10.35%	0	0	0	0.33%	0.38%	0
*Hirsutella*	0	0	0	0	0	0	0	0
*Lecanicillium*	0	0.53%	0	0	0	0	0.28%	0
*Metacordyceps*	0	0	0	0.02%	0	0	0	0.14%
*Metarhizium*	0.03%	<0.01%	0.11%	0	0	0	0	0
*Microsporum*	0.07%	0	0	0	0.14%	0	0	0
*Pochonia*	0.06%	0.06%	0	0	0	0	0.24%	0
*Purpureocillium*	0	0	<0.01%	0	0	0.16%	0	0
*Septobasidium*	0	0	0	0	0	0	0	0
*Simplicillium*	0.06%	2.20%	0	0	0	0.35%	0	0
*Trichosporon*	<0.01%	0	0.16%	20.52%	1.83%	0.54%	0	0
